# Rapid colorimetric loop-mediated isothermal amplification for hypersensitive point-of-care *Staphylococcus aureus* enterotoxin A gene detection in milk and pork products

**DOI:** 10.1038/s41598-020-64710-0

**Published:** 2020-05-08

**Authors:** Grittaya Srimongkol, Boonsong Ditmangklo, Ilada Choopara, Jiraporn Thaniyavarn, Deborah Dean, Sirirat Kokpol, Tirayut Vilaivan, Naraporn Somboonna

**Affiliations:** 1 Program in Biotechnology, Faculty of Science, Chulalongkorn University, Bangkok, 10330 Thailand; 2 Department of Chemistry, Faculty of Science, Chulalongkorn University, Bangkok, 10330 Thailand; 3 Department of Microbiology, Faculty of Science, Chulalongkorn University, Bangkok, 10330 Thailand; 4 Center for Immunobiology and Vaccine Development, UCSF Benioff Children’s Hospital Oakland Research Institute, Oakland, CA 94609 USA; 5 Department of Medicine, University of California, San Francisco, CA 94143 USA; 6 UC Berkeley/UCSF Graduate Program in Bioengineering, University of California, Berkeley, CA 94720 USA

**Keywords:** Assay systems, Applied microbiology

## Abstract

*Staphylococcus aureus* strains carrying enterotoxin A gene (*sea*) causes food poisoning and cannot be distinguished from non-pathogenic strains by the culture method. Here, we developed a rapid, specific and sensitive visual detection of *sea* using loop-mediated isothermal amplification (LAMP) combined with nanogold probe (AuNP) or styryl dye (STR). LAMP-AuNP and LAMP-STR can detect as low as 9.7 fg (3.2 *sea* copies) and 7.2 *sea* copies, respectively, which were lower than PCR (97 fg or 32 *sea* copies). The excellent performance of these new assays was demonstrated in food samples using crude DNA lysates. While the culture method detected 10^4^ CFU/g in ground pork and 10 CFU/mL in milk in 5–7 days, LAMP-AuNP could detect down to 10 CFU/g for both samples in 27 minutes. Analyzing 80 pork and milk samples revealed that the LAMP-AuNP showed 100% sensitivity, 97–100% specificity and 97.5–100% accuracy, which were superior to the culture method, and comparable to PCR but without requirement of a thermal cycler. Furthermore, our LAMP-AuNP detect *sea* at a range below the food safety control (<100 CFU/g). The LAMP-STR quantitated *sea* in 10–1,000 CFU (7.2–720 copies). Our crude DNA lysis combined with LAMP-AuNP/STR present effective point-of-care detection and facilitate appropriate control strategies.

## Introduction

*Staphylococcus aureus* strains with the staphylococcal enterotoxin A gene (*sea*) bacteriophage, are a common cause of foodborne disease and outbreaks worldwide including the United States and countries of the European Union^1–5^. In Thailand, one of the largest outbreaks occurred in 2009 reporting 573 illnesses due to consumption of contaminated sticky rice custard^6^. *S. aureus* is commonly found in soil, water, air, as well as human respiratory tract and skin. The *sea* toxin is relatively heat stable (e.g. up to 100 °C for 30 min) therefore, food contamination generally occurs when cooking is performed without sufficient hygiene. Frequently reported contaminated foods include dairy products (milk, cheese, ice cream, etc.), and meat^7,8^. Importantly, contaminated food may not be easily identified by sensory evaluation such as odor or appearance. Clinical symptoms from *sea* food poisoning include vomiting, abdominal cramps, diarrhea, and dysentery, often within 1–6 h after consumption. Occasional symptoms include sweating, chills, rectal bleeding, and shock^9–11^. The severity of illness depends on the concentration of the toxin. Consumption of merely 20–100 ng of *sea* toxin can cause clinical symptoms and >100 ng may be fatal, therefore a sensitive method of *sea* detection is important for food safety control. Detection is usually performed using culture based on a selective Baird-Parker medium^12^, with a limit of <100 bacterial colony forming unit (CFU) per g or mL of food or drink^13–15^. Nonetheless, this culture method supports both the growth of *sea* and non-*sea* strains, and thus lacks specificity in detecting the pathogenic *sea* strains. In addition, some food ingredients can affect the ability to culture leading to false negative results.

An alternative method for highly specific analysis of a food-borne pathogen is the nucleic acid-based assay, which generally requires amplification prior to detection to achieve the desired sensitivity. LAMP presents an attractive alternative to traditional amplification methods like PCR due to its low cost, ease-of-use and rapidity. Most importantly, the expensive thermal cycler and specialized kits for DNA extraction and purification are not required. The strand displacement characteristic of the *Bst* DNA polymerase and the loop-intercalating primers allow amplification of the targets at a single temperature with high sensitivity (e.g. 10–100 genome copies). The product can be visualized, albeit indirectly, by the formation of magnesium pyrophosphate as a white precipitate^16^. Goto *et al*.^2^. has previously developed a LAMP technique to detect *sea*, *seb*, *sec* and *sed* genes, and reported that it is more sensitive and specific than PCR by an order of magnitude^2,17^. However, previous analyses of LAMP results indirectly performed by monitoring the formation of magnesium pyrophosphate precipitate showed limited accuracy since precipitate formation affects reading accuracy. The alternative analysis by agarose gel-electrophoresis requires electrophoresis instrumentation, extra laboratory time (0.5–1.5 h), safety issues due to exposure to high voltage and ethidium bromide, and perhaps false positives due to misamplification^18^. Here, we present the use of a nanogold-appended *sea* probe (AuNP) specific for the target DNA to eliminate false positives, and increase assay specificity^19–24^. The high molar extinction coefficient of the AuNP supports simple visualization by naked eyes. Together, we designed new LAMP primers based on additional *sea* sequences from GenBank (https://www.ncbi.nlm.nih.gov/nucleotide/) along with two inner loop primers that can speed up the amplification by parallel-amplifying DNA loops outward on both ends^25^ and determined the optimal reaction conditions. The LAMP-AuNP assay was validated by testing commonly contaminated food samples (ground pork and milk) to show improved specificity and sensitivity^26–28^ compared to the standard nucleic acid-based assay (PCR). We also demonstrated that the combination of a simple crude DNA lysis method and LAMP is appropriate for point-of-care and resource-restrained diagnostics. Further, since the visual reading from LAMP-AuNP cannot quantitate the number of bacterial CFU (or copy), which is important when the contamination level is below the food and drink safety control (<100 CFU) but which the hypersensitive LAMP can detect^26,29^, an alternative detection method employing a cationic styryl dye (STR) as a fluorescent DNA stain^30,31^ was developed for quantifying the LAMP product. The LAMP-STR can quantify *sea* levels below 100 CFU, which a conventional culture method could not detect. Our two methods, therefore, help improve early detection of contaminated meat and dairy products to mitigate outbreaks of food poisoning.

## Materials and Methods

### Bacteria strains and culture

All bacteria strains were provided by the Department of Microbiology, Faculty of Science, Chulalongkorn University; Department of Medical Sciences, Ministry of Public Health; and Bamrasnaradura Infectious Diseases Institute, Ministry of Public Health (Supplemental Table 1). *S. aureus* ATCC 13565 and ATCC 25923 strains contain *sea*, representing a positive control. All other *S. aureus* strains and other bacteria species contain no *sea* and were used as negative controls. All bacteria strains were cultured in tryptic soy broth (Himedia, Mumbai, India) at 37 °C^17^.

### DNA extraction by commercial kit

GF-1 Bacterial DNA Extraction Kit (Vivantis, California, USA) was used to extract DNA from bacteria culture for preparation of strains, and from ground pork and milk samples for PCR. Following manufacturer’s protocols, 1 mL of bacterial cultures or 1 mL of aqueous extracts from minced ground pork (1 g/ 9 mL ddH_2_O) was centrifuged at 13,000 rpm 30 min at 4 °C to pelletize the bacterial cells. The pellet was then resuspended in Buffer R1 (Vivantis), and lysozyme and proteinase K were added to lyse bacterial cell membranes. Purified DNA was eluted in 50–100 µL of elution buffer (10 mM Tris-Cl, pH 8.5) and stored at −20 °C.

### S. aureus sea PCR

The PCR reaction for *S. aurFigure eus sea* (15 µL) comprised 12.5 µL EmeraldAmp® GT PCR Master Mix (TakaRa Bio, Shiga, Japan), 0.3 µM primers SEA-F and SEA-R (Supplemental Table 2), and 100 ng DNA (unless specified). Thermocycling conditions were as follows: 94 °C 2 min, followed by 35 cycles of 94 °C 2 s, 55 °C 2 min and 72 °C 1 min, and final extension at 72 °C 7 min^32,33^. The PCR product was analyzed by 1.75% agarose gel electrophoresis (120 base pairs (bp) amplicon).

### *S. aureus sea* LAMP assays by Goto *et al*.^2^ and our designed primers

Our designed LAMP primers were based on multiple sequence alignment of *sea* and non-*sea* sequences from GenBank. The 11 *sea* sequences were ATCC13565 (GenBank accession no. M18970), ATCC25923 (EF520720.1), MW2 (NC003923.1), TW20 (FN433596.1), phiNM3 (DQ530361.1), phiSa119 (KJ596420.1), Z172 (CP006838.1), MSSA476 (BX571857.1), MRSA252 (BX571856.1), ST228 (HE579073.1), and T0131 (CP002643.1). Primer Explorer V4 (Eiken Chemical, Tokyo, Japan; http://primerexplorer.jp/elampn4.0.0/index.htmL) along with manual design were performed. The primers specificity was validated by BLASTN against the GenBank nucleotide database. The sequences of primers from Goto *et al*.^2^ and our designed LAMP primers were compared (Supplemental 2). The LAMP reaction (15 µL) comprised 1.6 µM each of primers FIP, BIP, LF and LB, 0.2 µM primers F3 and B3, 1.4 mM dNTP (SibEnzyme Ltd., Novosibirsk, Russia), 0.3 M betaine (Sigma-Aldrich, St. Louis, MO, USA), 6 mM MgSO_4_, 8 U *Bst* DNA polymerase (New England Biolabs Inc., MA, USA), 1× ThermoPol^TM^ Reaction Buffer (New England Biolabs Inc.), and 100 ng DNA (unless specified). The reaction conditions (shortest incubation time with highest sensitivity and correct specificity) were optimized using incubation temperatures of 60, 63 and 65 °C, and incubation periods of 15, 30, 45 and 60 min. Heating at 80 °C for 2 min was used to terminate the reaction. The LAMP products appeared as intercalating bands of various bp sizes by 1.75% agarose gel electrophoresis.

### Preparation of nanogold probe and optimization for our LAMP-AuNP

The Probe-Sa_SEA (Supplemental Table 2) used for LAMP product detection was manually designed, and checked for specificity by BLASTN against the GenBank nucleotide database. Gold nanoparticles of 20 nm in diameter (Sigma-Aldrich) were appended to the Probe-Sa_SEA following methods of Jaroenram *et al*.^23^ and Somboonna *et al*.^22^. In brief, 50 µL of the Probe-Sa_SEA and 10 mL of gold nanoparticles were hybridized at 150 rpm at 50 °C in a hybridization oven (Thermo Scientific, New Jersey, USA), for 22 h. Next, 1 mL of 100 mM PBS, 10 µL of 10% SDS and 500 µL of 2 M NaCl, at 50 °C, were added, and the mixture was hybridized at 150 rpm at 50 °C for another 4 h. The mixture was centrifuged at 13,000 rpm 4 °C for 30 min. The clear solution was removed, and the precipitate was resuspended in 10 mM phosphate buffer (100 mM NaCl and 0.01% (w/v) SDS), and centrifuged again. The pellet was finally resuspended in 10 mM phosphate buffer (100 mM NaCl and 0.01% (w/v) SDS) to give the gold nanoparticles-probe solution (AuNP) an absorbance of 0.3–0.4 at 525 nm, and was stored protected from light at 4 °C. To determine the optimal conditions for the colorimetric LAMP-AuNP reaction, mixtures with different volume ratios of LAMP:AuNP (1:9 to 9:1) (10 μL total volume) were incubated at 63 °C for 10 min, and 0.01–1 M MgSO_4_ was then added. When a sufficient concentration of Mg^2+^ was present in the absence of the LAMP product, the AuNP agglomerated and changed its color. However, the positive LAMP products hybridize with the AuNP via their specific complementary strands, and the agglomeration induced by the salt addition was inhibited^20,22,24^. The LAMP-AuNP results could be interpreted by naked eyes via their color: red/purple (positive) or clear/very light purple (negative), which could be further confirmed by UV-vis spectrophotometry. Controls included AuNP only, AuNP with MgSO_4_, and no template control (DNA was replaced by ddH_2_O). The AuNP only displayed the same color as the positive LAMP results, while the AuNP with salt and no template control with salt showed the same color as the negative LAMP results. All controls showed the expected colors for all tests (data not shown).

### Comparison of specificity and sensitivity among *S. aureus sea* PCR, LAMP, and LAMP-AuNP methods

LAMP assays by Goto *et al*.^2^ (60 °C 30 min), as well as our LAMP (63 °C 15 min) and LAMP-AuNP (5:5 vol./vol.) were carried out at optimal conditions. Bacterial strains including *S. aureus* (*sea*), *S. aureus* (no *sea*), and other food poisoning bacteria (Supplemental Table 1), were tested for specificity. No template control referred to the LAMP reaction where DNA was replaced by ddH_2_O. For sensitivity, 10-fold serial dilutions of *S. aureus* (*sea*) DNA, or from live culture, were used as a template to determine the minimum limit of detection.

### Crude DNA lysis for ground pork and milk

Crude DNA lysis protocols for ground pork and milk were adapted from Reischl *et al*.^34^. and Sowmya *et al*.^35^. For ground pork and milk, the sample was stirred in a pellet pestle stirrer, and 1 mL of the liquid suspension was aliquoted into a microcentrifuge tube and centrifuged at 13,000 rpm 10 min. The supernatant was discarded, the pellet was suspended in 100 µL 1% Triton X-100 (Sigma-aldrich), heated at 95 °C 10 min, centrifuged at 13,000 rpm 10 min, and the supernatant was used as a DNA template for LAMP. The supernatant was stored at −20 °C.

### Comparison of specificity and sensitivity for *S. aureus sea* assays between culture and crude DNA lysis coupled with LAMP-AuNP methods using ground pork and milk spiked-in live bacteria culture

*S. aureus sea* strains ATCC13565 and ATCC25923 were cultured until an OD_600_ equal to 1 (10^8^ CFU) was obtained^17^. Samples with 10-fold serial dilutions were prepared ranging from 10^5^ to 10 CFU/mL, and 1 mL of each was spiked-in and mixed with 1 g pork or 1 mL milk samples, respectively. The samples were previously autoclaved and verified *sea*-free by *sea* PCR. For the pork samples, 9 mL of ddH_2_O were further added. For the culture assay, samples were analyzed by a certified ISO/IEC 17025 laboratory, Food Research and Testing Laboratory (FRTL, Faculty of Science, Chulalongkorn University), following the FDA’s Bacteriological Analytical Manual (BAM) for *S. aureus* protocols (https://www.fda.gov/food/laboratory-methods-food/bam-staphylococcus-aureus) that include: cultivation on selective Baird-Parker agar, followed by biochemical tests (oxidase test, catalase test, coagulase test and mannitol salt agar growth, and *S. aureus* should result catalase-positive and the rests are negative). For the LAMP-AuNP assay, the DNA was crude lysis, amplified and detected according to the protocols described above.

### Study design to compare the performance between the culture and our crude DNA lysis coupled with LAMP-AuNP assays

To determine the performance of the culture and our crude DNA lysis coupled with LAMP-AuNP methods, the extraction of DNA with GF-1 Bacterial DNA Extraction Kit (Vivantis) followed by *sea* PCR was used as the reference method. Statistically required sample numbers (N) were calculated according to the equation: N = (p (1-p) z^2^)/e^2^, given p at an average incidence of 18% (reported by Food Sanitation Division, Department of Health, in 2007 and 2008^36^, and Bureau of Epidemiology, in 2009^3^), z score of 1.65 for 90% confidence interval, and e of 10% for margin of error. This yielded an N of 40.18. Therefore, we used 40 ground pork and 40 milk samples purchased from local markets in Bangkok (Samyan and Kingpetch districts) and nearby provinces (Nakhon Pathom and Suphan Buri). The assay efficacy was calculated based on the following equations: sensitivity = true positive ÷ (true positive + false negative), specificity = true negative ÷ (true negative + false positive), false positive = 1 − specificity, false negative = 1 − sensitivity, and accuracy = (true positive + true negative) ÷ total samples. Verification of all samples results were based on results of a minimum of two independently confirmed GF-1 Bacterial DNA Extraction Kit (Vivantis) followed *sea* PCR^30^.

### LAMP-STR for colorimetric quantitation

The optimization experiments for LAMP-STR included testing different styryl dyes structures^37^, different dilutions, and different LAMP product diluent-to-STR diluent ratios (vol./vol.), that yielded the best fit data (R^2^, goodness of fit) for the regression equation analysis. In brief, the fluorescent DNA-binding styryl dye stock solution (20 µM) was diluted in phosphate buffer (pH 7.0); the LAMP product was diluted in double distilled water, then the appropriate LAMP-to-STR ratio was mixed by vortexing, the fluorescent red color (positive) result was visualized under a 315 nm UV light and a photograph was taken. ImageJ software (National Institutes of Health, Bethesda, Maryland, USA, https://imagej.nih.gov/ij/) was used to convert the image into numerical data by determining the color intensity from the fluorescent image.

## Results

### Optimal isothermal conditions of *sea* LAMP assays by Goto *et al*. and our designed primers

**Figure 1:**
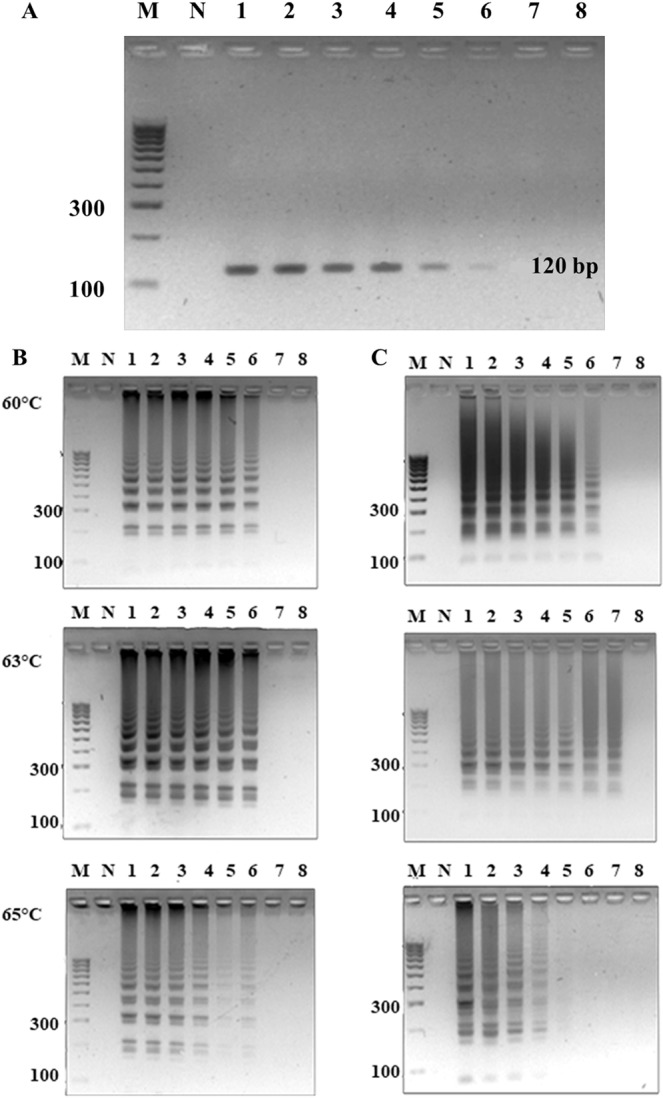
Limit of *sea* detection by (**A**) PCR, (**B**) Goto’s LAMP incubation period 30 min and temperatures 60, 63 and 65 °C, and (**C**) our LAMP incubation period 15 min and temperatures 60, 63 and 65 °C, using *S. aureus* ATCC13565 as template. Lane M represents 100 bp DNA ladder (GeneDireX Inc.); N, no template control; 1, 9.7 ng; 2, 0.97 ng; 3, 0.097 ng; 4, 9.7 pg; 5, 0.97 pg; 6, 0.097 pg; 7, 9.7 fg (equivalent to 3.2 *sea* copies); 8, 0.97 fg.

**Figure 2:**
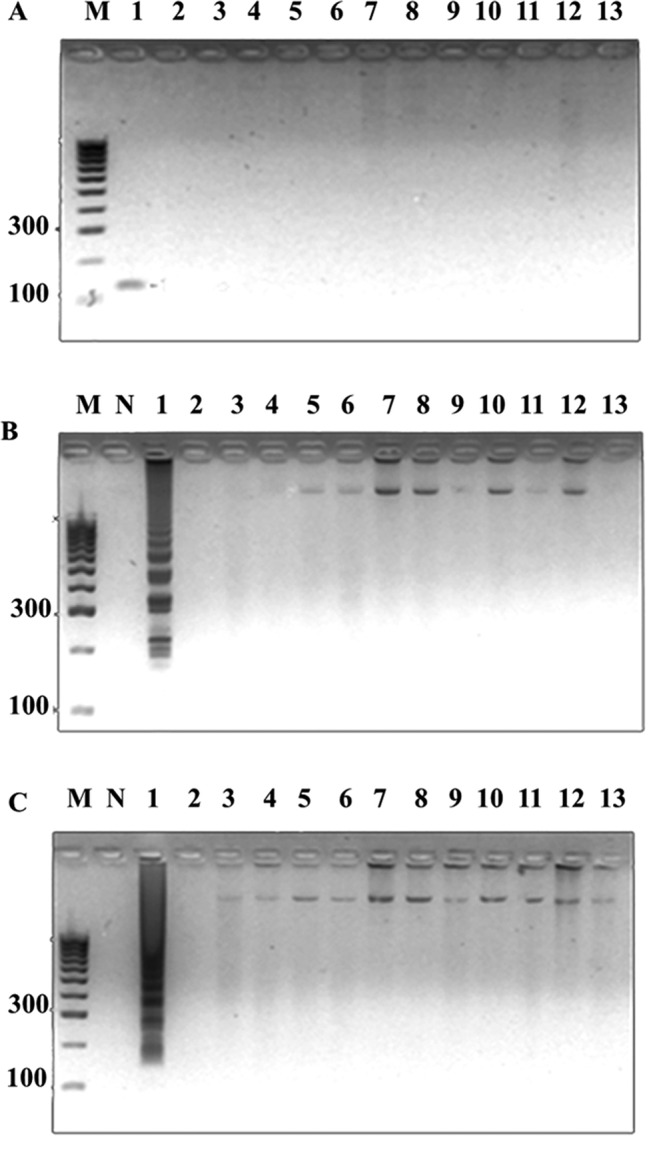
Specificity of *sea* detection by (**A**) PCR, (**B**) Goto’s optimal LAMP condition (60 °C 30 min), and (**C**) our LAMP optimal condition (63 °C 15 min). Lane M represents 100 bp DNA ladder (GeneDireX Inc.); N, no template control; 1, *S. aureus* ATCC13565; 2, *S. aureus* ATCC25928; 3, *S. aureus* ATCC144925; 4, *S. epidermidis* ATCC12228; 5, *S. saprophyticus* ATCC15305; 6, *V. funissii*; 7, *S. flexneri*; 8, *E. coli* ATCC25922; 9, *E. coli* ATCC35218; 10, *S. typhimurium* ATCC14026; 11, *E. cloacae*; 12, *A. sobria*; 13, *E. faecalis* ATCC25912.

Conditions were optimized over a temperature range of 60–65 °C and incubation periods of 15–60 min using 100 ng genome of *S. aureus* strains *sea* as template, and our designed LAMP primers that consisted of two inner loop primers (Supplemental Table 2, Sa_SEA LF and Sa_SEA LB) versus Goto et al.’s LAMP primers^2^ that contain only one inner loop primer (SEA_LB). Our LAMP primers showed LAMP products as early as 15 min at 60, 63 and 65 °C incubations. Meanwhile, Goto *et al*.’s LAMP primers earliest observable amplification was shown at 30 min (Supplemental Fig. 1). Noted without inner loop primers, both Goto *et al*.’s and our LAMPs showed the same optimal isothermal condition at 30 min and 65 °C (data not shown). The tentative isothermal conditions (15 min, and 60, 63 or 65 °C for our LAMP; 30 min, and 60, 63 or 65 °C for Goto *et al*.’s LAMP) were further selected based on specificity and sensitivity evaluations. A total of 14 foodborne bacterial strains (Supplemental Table 1, 2 *sea* and 12 non- *sea*) served as standard controls for *sea* PCR^32^, as well as Goto et al.’s and our LAMP specificity evaluation. All methods could correctly identify positive (PCR product appeared as a single band at ~120 bp, LAMP product appeared as multiple bands of different sizes) and negative control strains (showed no bands) (data not shown). This confirms the BLASTN results that our LAMP primer sequences were specific to only *S. aureus sea* (see Materials and Methods, details on the primer design requirement). For sensitivity determination, a 10-fold serial dilution containing 9.7 ng to 0.97 fg of *S. aureus* ATCC13565 genome served as templates. We selected 30 min at 63 °C as the isothermal condition for Goto *et al*.’s LAMP, and 15 min at 63 °C for our LAMP since these conditions require the smallest amount of template for successful amplification in each case. Hence, the detection limit of *sea* PCR and Goto *et al*.’s LAMP were found to be 0.097 pg (32 *sea* copies) while our LAMP was 9.7 fg (Fig. 1). The limit of detection results suggested a 10-fold increase in sensitivity for our LAMP compared to both PCR and Goto et al.’s LAMP. Proper specificity was found for all methods (Fig. 2).

### Specificity and sensitivity of our LAMP-AuNP

**Figure 3:**
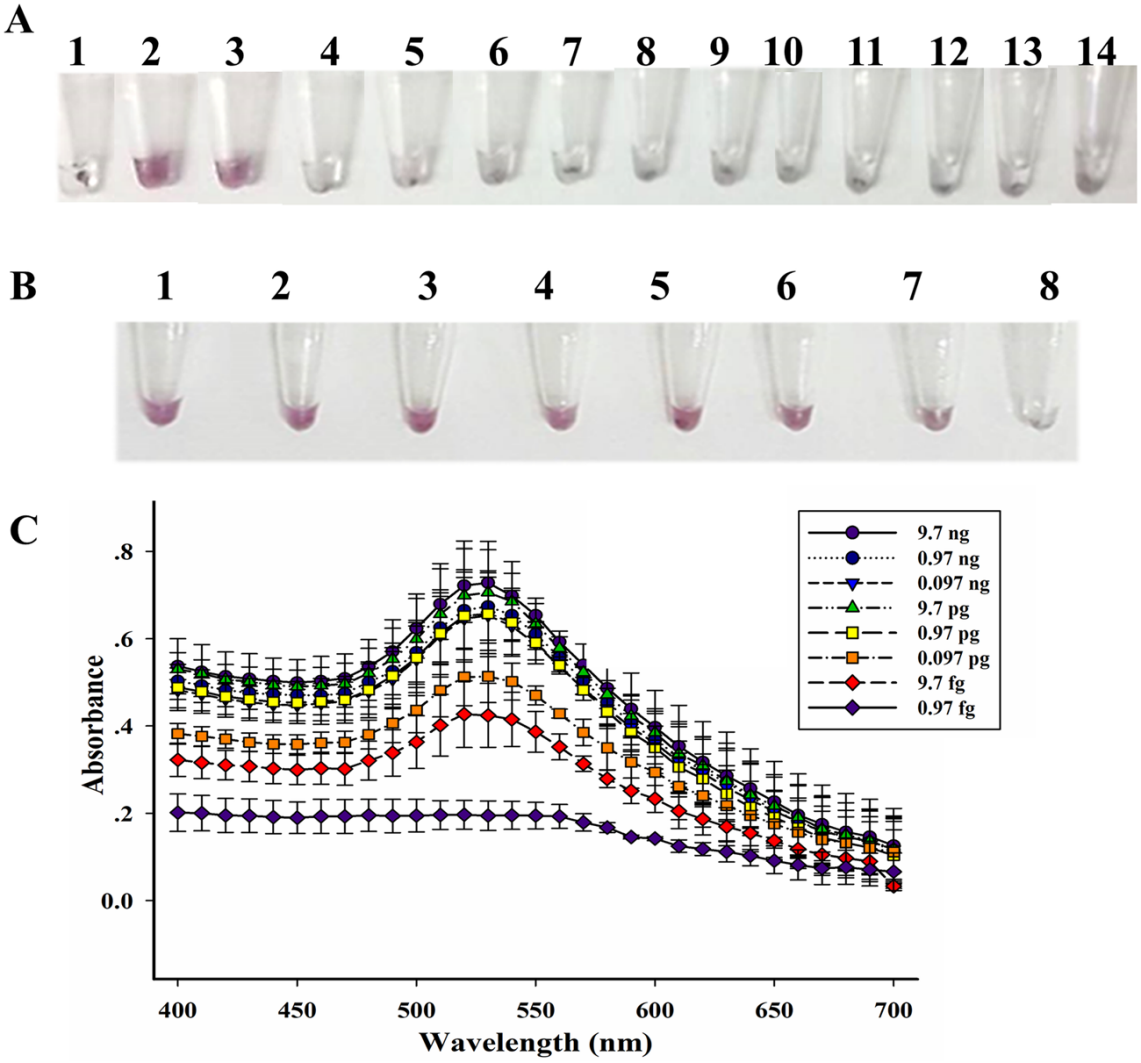
(**A**) Specificity, and sensitivity determined by (**B**) visual color and (**C**) spectrophotometry of LAMP-AuNP. In A, tube N represents no template control; 1, *S. aureus* ATCC13565; 2, *S. aureus* ATCC 25923; 3, *S. aureus* ATCC25928; 4, *S. aureus* ATCC144925; 5, *S. epidermidis* ATCC12228; 6, *S. saprophyticus* ATCC15305; 7, *V. funissii*; 8, *S. flexneri*; 9, *E. coli* ATCC25922; 10, *E. coli* ATCC35218; 11, *S. typhimurium* ATCC14026; 12, *E. cloacae*; 13, *A. sobria*; 14, *E. faecalis* ATCC25912. In B and C, *S. aureus* ATCC13565 DNA was as template; lane 1, 9.7 ng/µL; 2, 0.97 ng/µL; 3, 0.097 ng/µL; 4, 9.7 pg/µL; 5, 0.97 pg/µL; 6, 0.097 pg/µL; 7, 9.7 fg/µL; 8, 0.97 fg/µL.

Next, we attempted to combine our LAMP with AuNP probe to allow visual color detection through the specific complementary binding between the LAMP product and our designed *sea* oligonucleotide probe (Supplemental Table 1, Probe-Sa_SEA) attached to gold nanoparticles. The LAMP products corresponding to positive and negative samples were mixed with the AuNP probe at different ratios from 1:9 to 9:1 (vol./vol., total volume = 10 µL), and different amounts of salt (MgSO_4_); and the appropriate condition was selected where a significant color difference between positive (red/purple) and negative (clear/very light purple) could be clearly observed by the naked eye within 1–2 min. The optimal condition was found to be 5:5 of LAMP:AuNP (vol./vol., 5 µL/5 µL) and 5 µL of 0.5 M MgSO_4_. Noted that other ratios also caused the color change, but at a slower rate (up to 10 min), for examples the 3:7 and 4:6 LAMP:AuNP (vol./vol) (Supplemental Fig. 2). At a 1:9 ratio of LAMP:AuNP, the LAMP product was not present in sufficient amounts to inhibit the aggregation of the AuNP probe, while at too high amounts of the LAMP product (e.g. at 8:2 or 9:1), the color of the AuNP probe was difficult to observe. Hence, the ratio of 5:5 was most appropriate for the optimal detection since both positive and negative results could be judged relatively quickly within 1–2 min. The color difference as observed by the naked eye was confirmed by UV-vis spectrophotometry (wavelength scanning from 400 nm to 700 nm): positive peak at ~530 nm corresponded to red-purple color and negative results were observed as no peak (clear) or very minor peak (very light purple) (Supplemental Fig. 2, at 5:5 LAMP-to-AuNP). The appropriate LAMP:AuNP condition was used to evaluate specificity and sensitivity. Fourteen bacterial strains, in Supplemental Table 1, served as control for specificity evaluation, and our LAMP-AuNP correctly identified the positive and negative controls (Fig. 3A). In term of sensitivity, the LAMP-AuNP results as determined by the naked eye were fully consistent with our LAMP results as determined by agarose gel electrophoresis, showing a limit of detection as low as 9.7 fg, or equivalent to 3.2 *sea* copies (Figs. 1C and 3B). The color visualization of the LAMP-AuNP product was also further confirmed by UV-vis spectrophotometry (Fig. 3C)^20,21,24^.

### Crude DNA lysis coupled LAMP-AuNP assay is hypersensitive and effective in real ground pork and milk sample tests

We evaluated the feasibility of using the assay for the point-of-care diagnostics by utilizing DNA samples obtained from simple crude DNA lysis instead of from DNA extraction kits as a template for the LAMP-AuNP assay. In the crude DNA lysis, we compared different lysis buffer (TE Buffer^34^, 0.25 N NaCl and 0.0125% SDS^38^, and 1% Triton X-100^36^) from a minimum of three independent experiments and found that 1% Triton X-100 gave the highest DNA yield as determined by Nanodrop spectrophotometer. The average yield of crude DNA extracted by TE buffer, 0.25 N NaCl and 0.0125% SDS, and 1% Triton X-100 was 1.34, 1.68 and 1.99 ng/µL, respectively. Therefore 1% Triton X-100 was used for the preparation of the crude DNA lysates. Live cultures of individual bacterial strains in Supplemental Table 1 were used to spike ground pork at 10^5^ CFU/g and milk at 10^5^ CFU/mL, and our crude DNA lysis coupled with LAMP-AuNP assay was evaluated for specificity in comparison to the culture method due to its wide use and acceptance as an ISO/IEC 17025 standard assay for *S. aureus* diagnostics in local settings in Thailand. Our method correctly identified positive and negative results for *S. aureus sea*, while the culture method showed positive results for all *S. aureus sea* and non-*sea* strains (Table 1). For sensitivity evaluation, ground pork and milk with 10–10^5^ CFU/g and CFU/mL of *S. aureus* (*sea*) were tested. The culture method could detect as low as 10^4^ CFU/g whereas our assay could detect as low as 10 CFU/g of *sea* in ground pork samples. For milk samples, both the culture and our assays could detect as low as 10 CFU/mL (Table 2). Here, food matrices affected the limit of detection to culture *S. aureus*. It should be noted that there were differences in the sample preparation for the two assays. To maintain the bacterial viability in culture, the sample preparation method for culture is simply a mixing step between ground pork and ddH_2_O to obtain a liquid suspension without the use of harsh reagents or heat, so that live *S. aureus* could remain in pork debris. On the other hand, the crude DNA lysate preparation for LAMP required a mild detergent (Triton X-100) and boiling.

**Table 1:** Specificity comparison between the culture method by ISO/IEC 17025 laboratory and our crude DNA lysis coupled LAMP-AuNP on ground pork and milk samples spiked with live individual bacterial strains at 10^5^ CFU per g of ground pork (or mL of milk).

Pork	Culture (CFU/g)	LAMP-AuNP	Milk	Culture (CFU/mL)	LAMP-AuNP
*S. aureus* ATCC13565	+ (3,000,000)	+	*S. aureus* ATCC13565	+ (360,000)	+
*S. aureus* ATCC25923	+ (<110,000,000)	+	*S. aureus* ATCC25923	+ (<110,000,000)	+
*S. aureus* ATCC25928	+ (<110,000,000)	−	*S. aureus* ATCC 25928	+ (<110,000,000)	−
*S. aureus* ATCC144925	+ (<110,000,000)	−	*S. aureus* ATCC144925	+ (<110,000,000)	−
*S. epidermidis* ATCC12228	+/− (<10)	−	*S. epidermidis* ATCC12228	+/− (<10)	−
*S. saprophyticus* ATCC15305	+/− (<10)	−	*S. saprophyticus* ATCC15305	+/− (<10)	−
*V. funissii*	+/− (<10)	−	*V. funissii*	+/− (<10)	−
*S. flexneri*	+/− (<10)	−	*S. flexneri*	+/− (<10)	−
*E. coli* ATCC25922	+/− (<10)	−	*E. coli* ATCC25922	+/− (<10)	−
*E. coli* ATCC35218	+/− (<10)	−	*E. coli* ATCC35218	+/− (<10)	−
*S. typhimurium* ATCC14026	+/− (<10)	−	*S. typhimurium* ATCC14026	+/− (<10)	−
*E. cloacae*	+/− (<10)	−	*E. cloacae*	+/− (<10)	−
*A. sobria*	+/− (<10)	−	*A. sobria*	+/− (<10)	−
*E. faecalis* ATCC25912	+/− (<10)	−	*E. faecalis* ATCC25912	+/− (<10)	−

Results of the culture method were CFU/g (ground pork) or CFU/mL (milk), and when the culture resulted <10 CFU the analysis was recorded as undetermined using “+/−”.

**Table 2:** Sensitivity comparison between the culture method by ISO/IEC 17025 laboratory (10–10^5^ CFU per g of ground pork, or mL of milk) and our crude DNA lysis coupled LAMP-AuNP (1–10^5^ CFU per g of ground pork, or mL of milk) using live *S. aureus* ATCC13565 culture spike.

Pork (CFU/g)	Culture (CFU/g)	LAMP-AuNP	Milk (CFU/mL)	Culture (CFU/mL)	LAMP-AuNP
10^5^	+ (3,000,000)	+	10^5^	+ (360,000)	+
10^4^	+ (320,000)	+	10^4^	+ (34,000)	+
10^3^	+/− (<10)	+	10^3^	+ (1,900)	+
10^2^	+/− (<10)	+	10^2^	+ (235)	+
10	+/− (<10)	+	10	+ (20)	+
1	N/A*	−	1	N/A	−

N/A represents data not available, because 1 CFU spiked-in experiment was not performed for the culture method.

Next, we determined the statistical efficacy of our assay, and compared it to the culture method. A total of 80 ground pork and milk samples were analyzed and the standard DNA-based diagnostic method (commercial DNA extraction kit (GF-1 Bacterial DNA Extraction Kit, Vivantis) and *sea* PCR^32^) was used as the reference. The validity of the commercial DNA extraction kit combined *sea* PCR^32^ assay was first verified with ground pork and milk samples spiked with *S. aureus sea* at 10^5^ CFU/g and 10^5^ CFU/mL, respectively, as well as with live cultures of individual bacterial strains employing a similar experimental setup as in Table 1. The reference method properly identified positive and negative samples in all cases. Our assay could correctly detect *sea* positive and negative ground pork samples, giving identical results to the reference method (100% sensitivity, 100% specificity, 0% false positive and false negative, and 100% accuracy). For milk samples, our assay showed one false-positive resulting in the decrease in specificity (97%) and accuracy (97.5%). Conversely, the culture assay showed only 0–40% sensitivity, 90–97% specificity and 75–90% accuracy (Table 3).

**Table 3:** Comparison of assay efficacies between the culture method by ISO/IEC 17025 laboratory and our crude DNA lysis coupled with LAMP-AuNP, against reference *sea* detection method (GF-1 Bacterial DNA Extraction Kit (Vivantis) followed by *sea* PCR)^24,30^ on 40 ground pork and 40 milk samples surveillance from local markets in Bangkok and nearby provinces.

Samples	Reference *sea* detection method (number)	Culture method		Crude DNA extraction coupled LAMP-AuNP	
		Positive	Negative	Positive	Negative
Pork	Positive (7)	0	7	7	0
	Negative (33)	3	30	0	33
	Sensitivity	0.00		1.00	
	Specificity	0.90		1.00	
	False Positive	0.10		0.00	
	False Negative	1.00		0.00	
	Accuracy	0.75		1.00	
Milk	Positive (5)	2	3	5	0
	Negative (35)	1	34	1	34
	Sensitivity	0.40		1.00	
	Specificity	0.97		0.97	
	False Positive	0.03		0.03	
	False Negative	0.60		0.00	
	Accuracy	0.90		0.975	

### LAMP-STR for *sea* colorimetric quantitation

**Figure 4:**
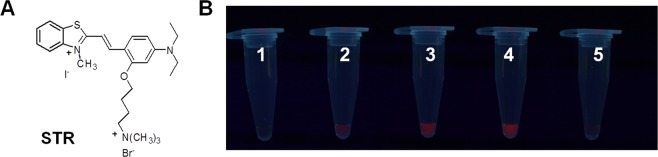
(**A**) Chemical structure of STR dye, and (**B**) photograph of STR dye (5 µM) with various LAMP samples at 80-fold dilution under UV light (315 nm). Tube 1 represents no LAMP product; 2–4 are LAMP products obtained from crude lysis DNA of *S. aureus* ATCC13565 (10, 100 and 1,000 CFU); 5, *E. coli* (1,000 CFU).

**Figure 5:**
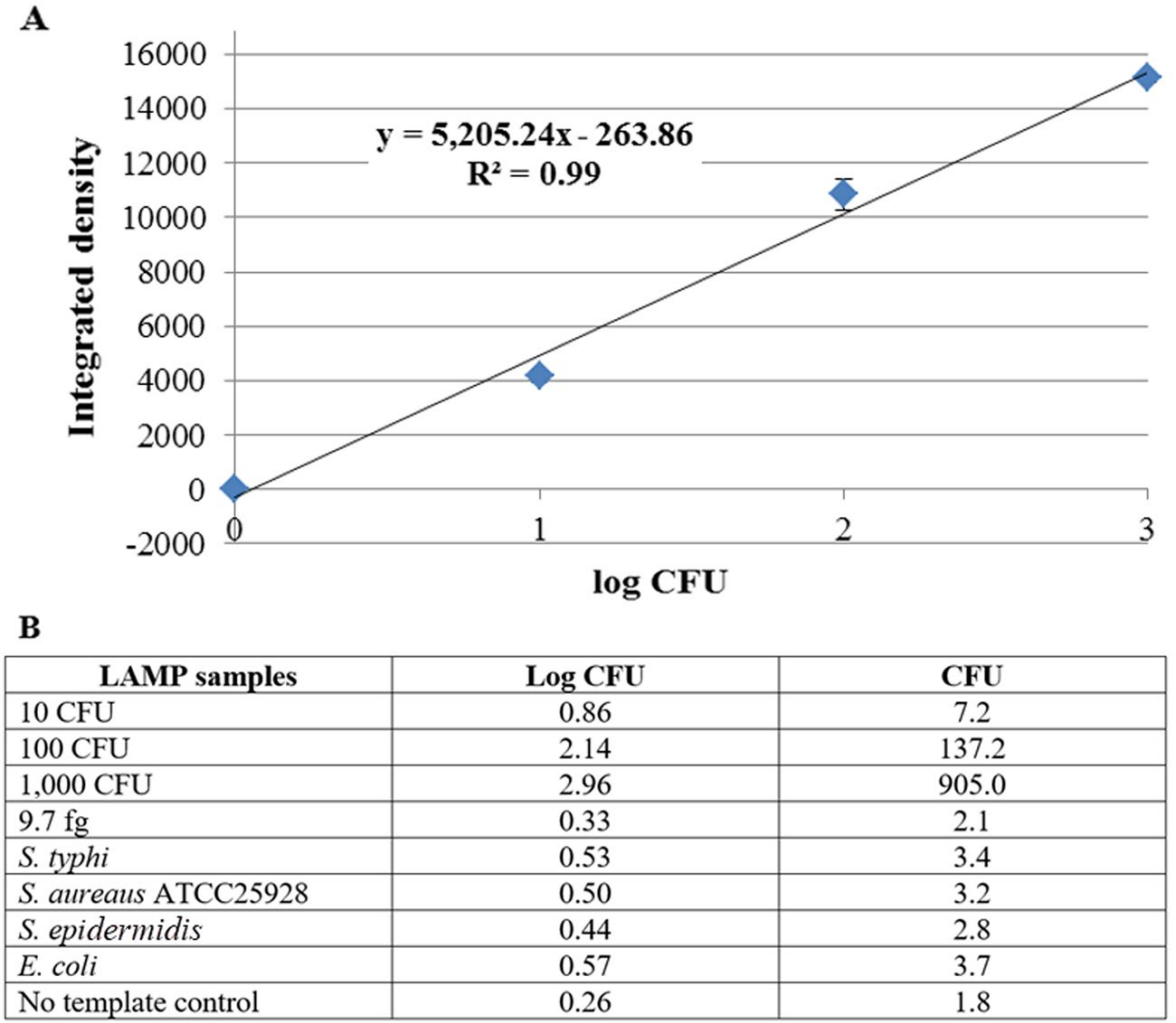
Accepted quantitative equation from 315 nm UV irradiation reading to CFU by (**A**) Goodness of fit (R^2^) of 0.99 from 5 independent experiments, and (**B**) blind testing with LAMP samples of known *S. aureus sea* concentrations (10, 100 and 1,000 CFU) and other bacterial species (negative).

The LAMP-AuNP method, while showing excellent specificity and sensitivity, could not easily quantitate the results. Small amounts of contamination may be present (e.g. <100 CFU), which could be a borderline case and can be acceptable under certain circumstances. However, a highly sensitive method that is qualitative in nature would simply indicate that such a sample is positive. Hence, there is a need for a method that allows quantitation of small amounts of DNA. In this context, we developed a new cationic fluorescent styryl dye (STR) (Fig. 4A) that can brighten up in the presence of DNA. Such dyes can be used in combination with the developed LAMP method to detect and quantitate low levels of LAMP product. Several styryl dyes were screened (data not shown), and only the cationic benzothiazolium dye designated as STR (see synthesis details in Supplementary Information^39^) was identified as the most appropriate dye that responded to the presence of LAMP product by showing bright orange fluorescence when visualized under UV light. At 5 µM of STR and 80-fold dilution of the LAMP product (Fig. 4B), the assay can correctly identify the *S. aureus sea* from negative controls (non-template and *E. coli*). Moreover, a different level of color intensity was observed at different amounts of the DNA template, suggesting the possibilities of performing a semi-quantitative analysis. A minimum of 5 independent experiments each containing 0, 10, 100 and 1,000 CFU LAMP products were thus analyzed and the linear calibration plot between the fluorescent intensity (Y variable) and the log CFU (X variable) was obtained (Fig. 5A). To confirm our derived quantitative equation, the live *S. aureus* (*sea*) culture (10, 100 and 1,000 CFU), the genomic DNA at the limit of detection of the LAMP-AuNP method (9.7 fg, or 3.2 copies), and the negative LAMP by the negative control strains or no template were evaluated. Our LAMP-STR was able to quantitate 10–1,000 CFU of *S. aureus* (*sea*), and for 3.2 copies LAMP the color intensity was found to be too small to be differentiated from the background density (Fig. 5B).

## Discussion

While not every enterotoxin type (such as type F) plays a significant role in food poisoning outbreaks in humans, the staphylococcal enterotoxin A represents the most commonly detected enterotoxin type associated with food poisoning outbreaks in humans, and having a minimal dose of infection of as little as 20 ng^40^. Staphylococcal enterotoxin A contamination is prevalent in meats, milk, eggs and foods high in fat. Conventional methods of detection involve culturing on selective Baird-Parker agar, in which *S. aureus* colonies should appear shiny black and circular surrounded by clear zone. *S. aureus* confirmation may be optionally performed by four biochemical tests (catalase, oxidase, coagulase and mannitol salt tests). Yet the culture method cannot differentiate *sea* from non-*sea* strains. This method is also labor and time intensive (2–4 days), expensive (>10 USD per test), and the sensitivity and specificity are limited compared to the DNA-based diagnostic. In DNA diagnostics, although PCR gives excellent performance in terms of sensitivity and specificity, the major limitation is its requirement of an expensive thermal cycler and a 3–4 h diagnostic time. This study developed a combined crude DNA lysis as a means for sample preparation with *S. aureus sea* LAMP-AuNP and LAMP-STR that offers similar, but accelerated (<1 h) performance compared to PCR, and required no thermal cycler or electrophoresis instruments. LAMP offers a simple, rapid and effective targeted amplification, while AuNP (visual color) and STR (fluorescent) offer easy color readouts.

The two inner loop primers, LF and LB, that we designed could accelerate the DNA amplification by producing loop amplicons from multiple sized LAMP amplicons from the inside outward to both ends. This increased the amount of LAMP product, so the assay time was reduced by half from 30 to 15 min compared to Goto *et al*.’s LAMP (and our LAMP without inner loop primers, data not shown). The limit of detection according to gel electrophoresis was also higher^20,23,24^ (from 97 fg to 9.7 fg genome, or equivalent 3.2 *sea* copies). To enable point-of-care and resource-constrained diagnosis, AuNP functionalized with a probe complementary to the LAMP product was employed to replace the agarose gel electrophoresis for detection of the specific LAMP products. This allows naked eye detection with high accuracy due to complementary binding between the target (in LAMP product) and the probe^41,42^. Moreover, since the *Bst* DNA polymerase used in LAMP is much less sensitive to inhibitions than the *Taq* DNA polymerase typically employed in PCR, the sample preparation can be greatly simplified. The crude DNA lysis from real food samples, which often contain inhibitors to the culturing or the *Taq* DNA polymerase in PCR^17,38,43,44^, was successfully employed. This study selected ground pork and milk as food samples, given that ground pork often had problems with the culture method, and both ground pork and milk required commercial DNA extraction kits for the PCR method. While the efficiency of the culture method was biased to sample types (poor limit of detection and assay accuracy for ground pork samples), our crude DNA lysis coupled with LAMP-AuNP showed a better limit of detection and a much higher assay accuracy for both ground pork and milk samples (10 CFU/g or mL, and 97.5–100% accuracy). Our assay accuracy was comparable to using commercial DNA extraction kit together with the PCR methods. Despite the fact that enterotoxin A accounts >50% of causes in some areas, the ability to detect every enterotoxin type gene that causes food poisoning is important. With appropriate primers and reaction conditions, our assay can be adapted for detecting and quantitating other enterotoxin type genes even with a minimal contamination.

Recently, several LAMP-based assays have been developed to detect foodborne pathogens, such as staphylococci^45^, *Listeria monocytogenes*^46^, *Vibrio parahaemolyticus*^47,48^, *Pseudomonas aeruginosa*^49^, *Salmonella*^50^ and *Escherichia coli*^51^. Whilst our research reported similarly high assay sensitivity and specificity, our LAMP-based assay directly detected the pathogenic *S. aureus* strain with *sea*, and compared assay efficiencies including detection limit, sensitivity, specificity, false positives, false negatives and accuracy, between culture methods and our crude DNA lysis coupled with LAMP-AuNP methods in both laboratory and real food sample tests with either spiked-in or natural *S. aureus sea* contaminations (statistical number of samples). Our assay also contained the double inner loop primers to allow a rapid 15 min LAMP reaction time, and had combined STR quantitation methods, making the detection time starting from raw sample to color readout data analysis within 1 h.

## Conclusion

The data supported the reliability of our assay for *S. aureus sea* without the requirement of a commercial DNA extraction kit nor expensive instruments like a thermal cycler, electrophoresis apparatus, or a spectrophotometer. Only a simple heat block or water bath and readily available reagents are sufficient to perform our hypersensitive and highly accurate diagnostic test at a point-of-care setting in less than 1 h (30 min crude sample lysis, 15 min LAMP, and 7 min AuNP steps) while requiring minimal expertise. Our assay was effective with various types of samples including DNA, live bacterial culture, pork and milk spiked with live bacterial culture, and throughout a statistical number of market sample surveillance (Thai petty patent no. 16030012151 for LAMP-AuNP). An additional assay, LAMP-STR, was developed to quantitate low levels of *S. aureus sea* contamination between 10–1,000 CFU (Thai petty patent no. 1703000138). This facilitates the case when <100 CFU/g or mL diet is acceptable, therefore LAMP-STR will allow proper management of diets with contamination but under the control standard.

## Supplementary information

Supplementary information.
