# Supplementary material for: Rapid colorimetric loop-mediated isothermal amplification for hypersensitive point-of-care Staphylococcus aureus enterotoxin A gene detection in milk and pork products
Source: Sci Rep. 2020 May 8;10:7768. doi: 10.1038/s41598-020-64710-0 (PMC7211006; doi:10.1038/s41598-020-64710-0)
Supplement: Supplementary file 1 — Supplementary information. [file 41598_2020_64710_MOESM1_ESM.pdf]

Rapid colorimetric loop-mediated isothermal amplification for hypersensitive point-of-care *Staphylococcus aureus* enterotoxin A gene detection in milk and pork products

Grittaya Srimongkol[a], Boonsong Ditmangklo[b], Ilada Choopara[a], Jiraporn Thaniyavarn[c], Deborah Dean[d,e,f], Sirirat Kokpol[b], Tirayut Vilaivan[b], and Naraporn Somboonna[c*]

Supplementary Figures and Tables

Supplemental Figure 1. Incubation periods and temperatures of (A-C) Goto's and (D-F) our LAMPs.

In (A and D) incubation temperature was at 60 °C, (B and E) 63 °C, and (C and F) 65 °C. Lane M represents 100 bp DNA ladder (GeneDireX Inc., Miaoli County, Taiwan); N, negative control; 1-4, incubation period at 15, 30, 45 and 60 min, respectively. (Original photographs of A-C is in Supplementary Information).

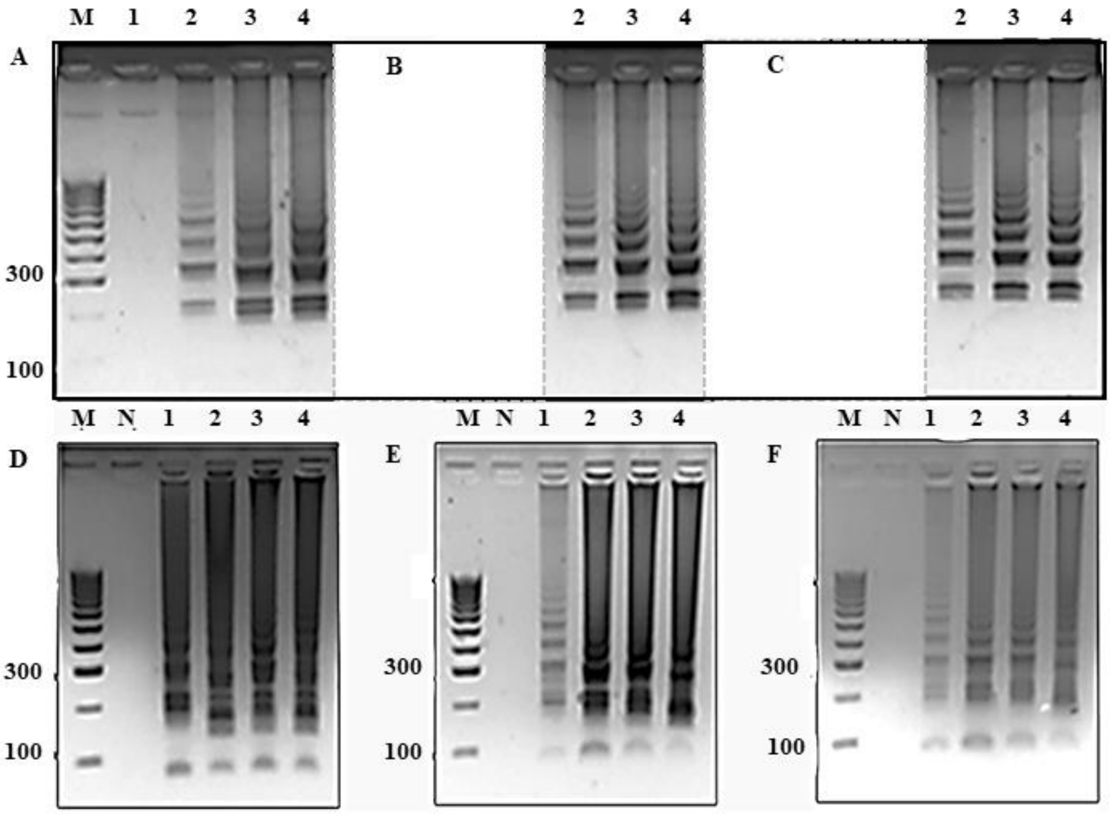

M 1 2 3 4
2 3 4
2 3 4
A
B
C
300
100
M N 1 2 3 4
M N 1 2 3 4
M N 1 2 3 4
D
E
F
300
100
300
100
300
100

Supplemental Figure 2. Optimized ratios of LAMP-to-AuNP volume for optimal color contrast between positive (red) and negative (purple/blue/gray) test results, determined by visualization and spectrophotometer at 400-700 nm absorbance. An optimal ratio was 5:5 LAMP:AuNP.

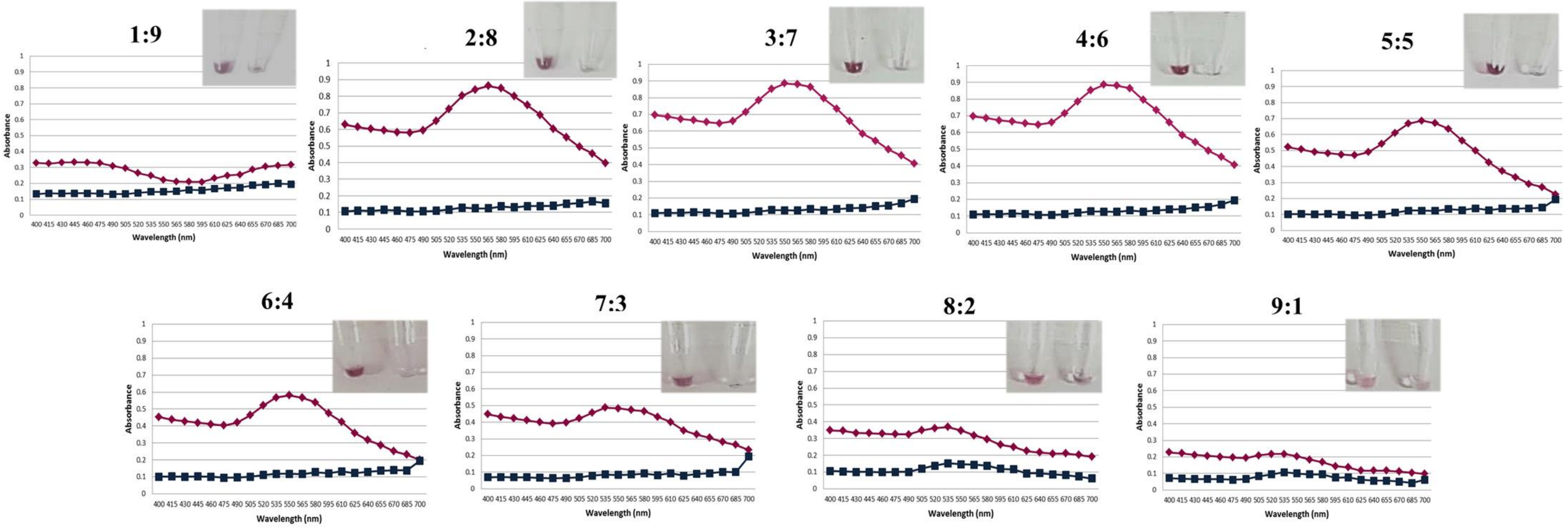
1:9
2:8
3:7
4:6
5:5
6:4
7:3
8:2
9:1
Absorbance
Wavelength (nm)

Supplemental Table 1. Bacterial strains.

Bacteria species	*Strain numbers*	*Sources*
Staphylococcus aureus (sea)<br>*Staphylococcus aureus*<br>*Staphylococcus aureus*	*ATCC13565*<br>*ATCC25928*<br>*ATCC144925*	*Department of Microbiology, Faculty of Science, Chulalongkorn University*
Staphylococcus aureus (sea)<br>*Staphylococcus epidermidis*<br>*Staphylococcus saprophyticus*	*ATCC25923*<br>*ATCC12228*<br>*ATCC15305*	*Department of Medical Sciences, Ministry of Public Health*
Vibrio funissii<br>*Shigella flexneri*<br>*Escherichia coli*<br>*Escherichia coli*<br>*Salmonella typhimurium*<br>*Enterobacter cloacae*<br>*Aeromonas sobria*<br>*Enterococcus faecalis*	*N/A**<br>*N/A*<br>*ATCC25922*<br>*ATCC35218*<br>*ATCC14026*<br>*N/A*<br>*N/A*<br>*N/A*	*Bamrasnaradura Infectious Diseases Institute, Ministry of Public Health*

* N/A represents information on ATCC number that is not available, because strain was isolated and sequenced to identify species at local source.

Supplemental Table 2. Oligonucleotide primer and probe sequences.

Reaction	***Primer and probe names***	***Sequences (5′- 3′)***
PCR[30]	*SEA-F*	*TTGGAAACGGTTAAAACGAA*
	SEA-R	*GAACCTTCCCATCAAAAACA*
Goto et al.'s LAMP[2]	*SEA_FIP*	*GATCCAACTCCTGAACAGTTACAATACAGTACCTTTGGAAACG*
	SEA_BIP	*CTGATGTTTTTGATGGGAAGGTTCCCGAAGGTTCTGTAGAAGT*
	SEA_F3	*TCAATTTATGGCTAGACGGT*
	SEA_B3	*CTTGAGCACCAAATAAATCG*
	SEA_LB	*AGAGGGGATTAATCGTGTTTCA*
Our LAMP	*Sa_SEA FIP*	*CTGTAAAAAAGCCTTTAAACAATATTTTGCTAAAACTGAAAATAAAGAGAGTC*
	Sa_SEA BIP	*GGTATAACGATTTATTAGTAGATTTTTATACAAGTCTACTTTTTTCCCTT*
	Sa_SEA F3	*TCTATTATTACAATGAAAA*
	Sa_SEA B3	*ATTGATAACCATAATAAGCA*
	Sa_SEA LF	*TGCTAGTTAAAAATGTCGTATGAT*
	Sa_SEA LB	*GATTCAAAGGATATTGTTGAT*
Our LAMP-AuNP	*Probe-Sa_SEA*	*HS-AGGCTTTTTTACA(G/A)ATCATTC*

Supplementary Information

Supplementary Information 1. Original photographs of Supplemental Figure 1A, 1B and 1C

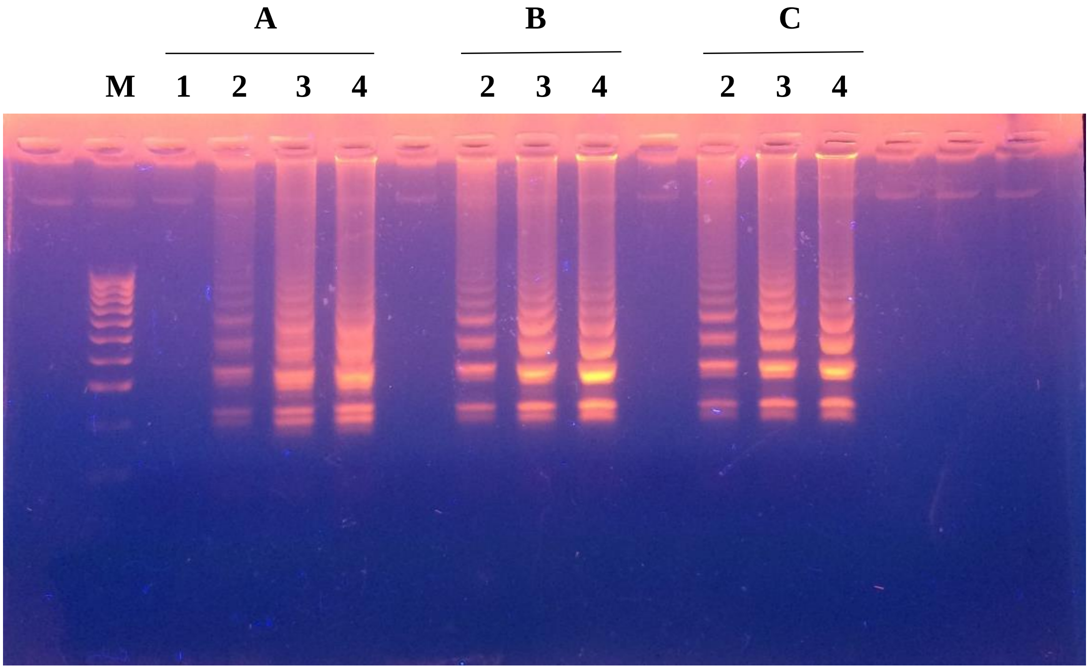

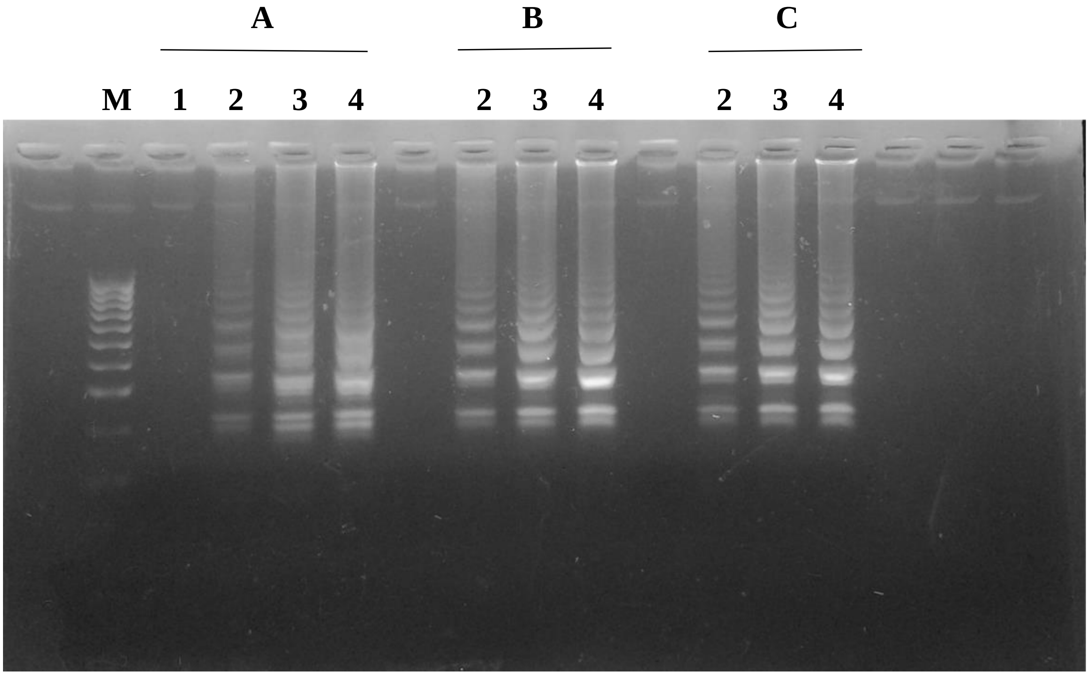

Supplementary Information 2. Synthesis of STR dye

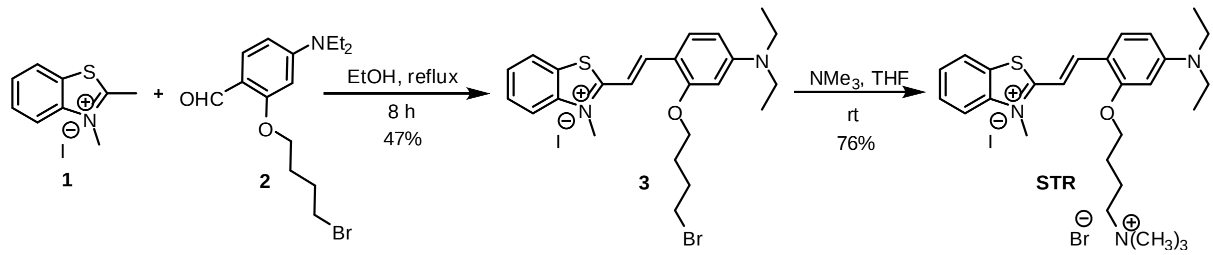

A sample of 2,3-dimethyl-1,3-benzothiazol-3-ium iodide (**1**) (80 mg, 0.3 mmol) and *2*-*(4*-bromobutoxy)-*4*-*(*diethylamino)-benzaldehyde (**2**) (200 mg, 0.6 mmol)[39] was refluxed in absolute ethanol for 8 h. The precipitate formed was recrystallized from ethanol to give the intermediate **3** as a gray solid (78 mg, 47%): ^{1}H NMR (400 MHz, DMSO-d_6): δ = 8.20 (d, J = 7.4 Hz, 1H), 8.08 (d, J = 14.5 Hz, 1H), 8.00 (d, J = 8.0 Hz, 1H), 7.90 (d, J = 9.2 Hz, 1H), 7.73 (t, J = 7.7 Hz, 1H), 7.62 (t, J = 7.25 Hz, 1H), 7.48 (d, J = 15.1 Hz, 1H), 6.51 (d, J = 8.74 Hz, 1H), 6.20 (s, 1H), 4.20 (t, J = 6.0 Hz, 2H), 4.11 (s, 3H), 3.65 (t, J = 6.3 Hz, 4H), 3.54 (m, 4H), 3.40 (t, J = 6.6 Hz, 1H), 2.00 (m, 4H), 1.15 (t, J = 6.9 Hz, 6H)

Trimethylamine (1 mL, 5 mmol) was added to a solution of **3** (30 mg, 0.05 mmol) in tetrahydrofuran (2 mL) and the reaction was stirred for 30 h at room temperature. The reddish-purple precipitate was filtered to give the dye **STR (**30 mg, 76%): ^{1}H NMR (400 MHz, DMSO-d_6): δ = 8.22 (d, J = 7.4 Hz, 1H), 8.10 (d, J = 14.5 Hz, 1H), 8.02 (d, J = 8.00 Hz, 1H), 7.90 (d, J = 9.2 Hz, 1H), 7.73 (t, J = 7.7 Hz, 1H), 7.62 (t, J = 7.2 Hz, 1H), 7.48 (d, J = 15.1 Hz, 1H), 6.51 (d, J = 8.7 Hz, 1H), 6.20 (s, 1H), 4.20 (t, J = 6.0 Hz, 2H), 4.11 (s, 3H), 3.51 (m, 4H), 3.44 (m, 2H), 3.09 (s, 9H), 1.89 (m, 4H), 1.15 (t, J = 6.9 Hz, 6H)

The dye **STR** exhibited maximum absorption at 525 nm and emission at 591 nm and the fluorescence emission increased by more than 18-fold in the presence of DNA.
